# Aspects épidémiologiques, cliniques et thérapeutiques des algies pelviennes aiguës de la femme à l’Hôpital Gynéco-Obstétrique et Pédiatrique de Yaoundé (HGOPY)

**DOI:** 10.11604/pamj.2020.35.16.14332

**Published:** 2020-01-23

**Authors:** Pierre-Marie Tebeu, Etienne Belinga, Julius Dohbit Sama, Sandrine Adeline Kenmogne, Charlotte Tchente, Isaac Sandjong

**Affiliations:** 1 Département de Gynécologie-Obstétrique, Faculté de Médecine et de Sciences Biomédicales, Université de Yaoundé I, Centre Hospitalier et Universitaire, Cameroun; 2 Ligue d’Initiative et de Recherche Active pour la Santé et l’Education de la Femme (LIRASEF), Yaoundé, Cameroun; 3 Département de Gynécologie-Obstétrique, Faculté de Médecine et de Sciences Biomédicales, Université de Yaoundé 1, Centre Hospitalier de Recherche et d’Application en Chirurgie Endoscopique et Reproduction Humaine, Yaoundé, Cameroun; 4 Département de Gynécologie-Obstétrique, Faculté de Médecine et des Sciences Biomédicales, Université de Yaoundé I, Hôpital Gynéco-Obstétrique et Pédiatrique de Yaoundé, Yaoundé, Cameroun; 5 Faculté de Médecine et des Sciences Pharmaceutiques, Université de Douala, Douala, Cameroun; 6 Service de Gynécologie Obstétrique, Hôpital Gynéco-Obstétrique et Pédiatrique de Yaoundé, Yaoundé, Cameroun

**Keywords:** Algies pelviennes aiguës, grossesse extra-utérine, infections génitales hautes, Yaoundé, Acute pelvic pain, ectopic pregnancy, upper genital infections, Yaoundé

## Abstract

**Introduction:**

Les algies pelviennes aiguës sont responsables d’une morbi-mortalité importante. L’objectif de ce travail était de décrire leurs aspects épidémiologiques, cliniques et thérapeutiques à Yaoundé

**Méthodes:**

Nous avons mené une étude transversale descriptive avec collecte prospective des données au Service de Gynécologie-Obstétrique de l’Hôpital Gynéco-Obstétrique et Pédiatrique de Yaoundé du 1^er^ avril au 31 juillet 2015. Nous avons inclus toutes les femmes admises pour douleur pelvienne dont l’évolution était inférieure à un mois et ayant accepté de participer à l’étude. Nous avons exclu les femmes qui étaient au troisième trimestre de grossesse ou en post-partum. Le logiciel Epi info version 3.5.4 a servi à l’analyse des données. Ces données ont été présentées sous forme de fréquence et de pourcentage.

**Résultats:**

Au total 5915 femmes ont consulté pendant la période de l’étude dont 125(2,11%) étaient des algies pelviennes aiguës. La moyenne d’âge était de 29,5 ± 6,9 ans. Les étiologies des douleurs étaient les infections génitales hautes (36,8%) et la grossesse extra-utérine (18,4%). Le traitement surtout médical (92,8%), associait antibiotiques (65,5%), anti-inflammatoires (56,9%) et antalgiques (39,7%). La chirurgie a été réalisée chez 25(20%) patientes par laparotomie (80%) et cœlioscopie (20%). L’indication chirurgicale était la grossesse extra-utérine dans 76% des cas. La régression de la douleur était obtenue chez 99% des cas.

**Conclusion:**

Les d’algies pelviennes aigues survenaient chez les femmes jeunes, dues aux infections génitales hautes et à la grossesse extra-utérine étaient principalement. En cas de grossesse extra-utérine le traitement chirurgical était surtout la laparotomie.

## Abstract (en)

**Introduction:**

Acute pelvic pain is an important cause of morbi-mortality. The purpose of this study was to describe the epidemiological, clinical and therapeutic features of acute pelvic pain in Yaoundé.

**Methods:**

We conducted a cross-sectional, descriptive study with collection of prospective data in the Department of Gynecology and Obstetrics at the Yaoundé Gynaecology-Obstetrics and Pediatrics Hospital over the period 1^st^ April-31^st^ July 2015. We included all female subjects admitted for pelvic pain whose course was less than one month and who agreed to participate in the study. All women who were in the third trimester of pregnancy or in the post-partum period were excluded. Epi info software, version 3.5.4 was used to analyze data. Data were shown as frequency and percentage.

**Results:**

During the study period, a total of 5915 women presented to the Yaoundé Gynaecology-Obstetrics and Pediatrics Hospital, of whom 125 (2.11%) had acute pelvic pain. The average age of patients was 29.5 ± 6.9 years. Pain was caused by upper genital tract infections (36.8%) and ectopic pregnancy (18.4%). Most patients received medical treatment (92.8%), associated with antibiotics in 65.5% of cases, anti-inflammatory drugs in 56.9% of cases and analgesics in 39.7% of cases. Surgery was performed in 25 (20%) patients via laparotomy (80%) and coelioscopy (20%). Surgery was indicated in patients with ectopic pregnancy (76% of cases). Regression of pain was obtained in 99% of cases.

**Conclusion:**

Acute pelvic pain mainly affected young women with upper genital tract infections and ectopic pregnancy. In the case of ectopic pregnancy surgical treatment via laparotomy was the gold standard treatment.

## Introduction

Les algies pelviennes aiguës ou douleurs pelviennes d’évolution récente (moins d’un mois) sont localisées au niveau de l’hypogastre et des fosses iliaques gauche et droite [1,2]. Plusieurs affections gynécologiques et non gynécologiques peuvent en être la cause. A Madagascar, l’incidence est de 15,5% [3]. L’absence de diagnostic précoce ou de traitement adapté peut entraîner une morbidité sévère y compris le pronostic de fertilité de la femme (maladie inflammatoire pelvienne, torsion d’annexe). Le retard au diagnostic et la prise en charge peuvent entrainer le décès de la femme (grossesse extra-utérine (GUE) rompue). Le clinicien en charge des urgences en gynécologie et obstétrique doit être prêt à reconnaitre les affections responsables d’algies pelviennes aigues [3,4]. Malgré la disponibilité des moyens diagnostiques et thérapeutiques et l’ampleur des conséquences dues aux algies pelviennes aigues chez nous, aucune donnée n’est disponible sur le sujet au Cameroun. L’objectif était de décrire les aspects épidémiologiques, cliniques et thérapeutiques des algies pelviennes aiguës de la femme à l’Hôpital Gynéco-Obstétrique et Pédiatrique de Yaoundé (HGOPY) au Cameroun.

## Méthodes

Il s’agit d’une étude transversale descriptive avec collecte prospective des données du 1^er^ avril au 31 juillet 2015 au Service de Gynécologie-Obstétrique de HGOPY. Nous avons inclus toutes les patientes qui présentaient une douleur pelvienne évoluant depuis moins de 30 jours et ayant consenti de participer à l’étude. Les femmes au troisième trimestre de grossesse ou en post partum étaient exclues. L’échantillonnage était consécutif en consultation externe et en hospitalisation. La collecte des données était anonyme sur une fiche préétablie et pré-testée après obtention de la clairance éthique auprès des autorités compétentes. Les données sociodémographiques, cliniques para cliniques et thérapeutiques constituaient nos variables d’intérêt. L’analyse des données s’est faite grâce au logiciel Epi info version 3.5.4. Les données quantitatives ont été présentées sous-formes de moyennes avec leurs écart-types et les données qualitatives sous-forme de fréquences et de pourcentages.

## Résultats

Au total, parmi les 5915 patientes ayant consulté durant la période de l’étude, 448(7,6%) avaient des algies pelviennes. Parmi elles 125 étaient des algies pelviennes aiguës (APA), soit une prévalence des APA de 2,11%. La moyenne d’âge des femmes était de 29,5 ± 6,9 ans. Les patientes étaient majoritairement étudiantes 31(24,8%) suivies des ménagères 30(24,0%). Elles pratiquaient surtout la religion catholique 87(69,6%) (Tableau 1). Plus de la moitié (53%) des patientes avaient un antécédent d’avortement. C’était surtout des avortements provoqués (66,7%). La méthode utilisée était surtout le curetage/aspiration (54,5%). Seulement 40,8% des femmes utilisaient les méthodes modernes de contraception. Elles utilisaient surtout le préservatif masculin (60,8%), la pilule (13,7%) et les injectables (11,8%). La quasi-totalité des patientes (93%) avaient un seul partenaire sexuel. Quatre patientes (3,2%) avaient un antécédent de grossesse extra-utérine traitée chirurgicalement. Le signe physique le plus observé était la douleur à la palpation profonde chez 60 patientes (48%). La douleur était soit localisée à la fosse iliaque droite chez 19 patientes (15,2%), soit à l’hypogastre chez 18 patientes (14,4%) ou alors localisée à la fosse iliaque gauche chez 11 patientes (8,8%). Le toucher vaginal était normal chez 13 patientes (10,4%). La mobilisation du col était douloureuse chez 77 patientes (61,6%).

**Tableau 1:** Répartition des patientes selon les caractéristiques sociodémographiques

Caractéristiques sociodémographiques	Algies pelviennes aigues total 125
	Effectif (n)	%
**Age moyen (ans)**	**29,5(DS+ 6,9)**	
**Profession**		
Etudiante	31	24,8
Ménagère	30	24,0
Autres*	64	51,2
**Réigion**		
**Catholique**	**87**	**69,6**
Protestante	27	21,6
Autres**	11	8,8
**Région d’origine**		
Centre	52	41,6
Ouest	46	36,8
Autres***	27	21,6
**Statut matrimonial**		
Célibataire	60	48,0
Mariée	58	46,4
Autres****	7	5,6
**Niveau d’instruction**		
**Supérieur**	**57**	**45,6**
**Secondaire**	**57**	**45,6**
Autres*****	11	8,8

* emploi rémunérénon spécifié ;

** Musulmane, pentecôtiste, adventiste ;

*** Littoral, Sud, Nord-Ouest, Sud-Ouest, Est, Adamaoua, Nord, Extrême Nord ;

**** Veuve, union libre ;

***** Primaire, non scolarisée

Les examens biologiques demandés étaient surtout: la numération formule sanguine (NFS) (43,2%), le prélèvement vaginal (PV) (33,6%), la sérologie chlamydiae (27,2%) et la recherche de mycoplasme urogénital (26,4%). Seules 40/54 patientes ont réellement fait la NFS. Les anomalies étaient surtout l’anémie (75,5%), l’hyperleucocytose (30%), la thrombopénie (17,5%). Au prélèvement vaginal, le germe isolé était surtout *Gardnerella vaginalis* (68,7%). Seulement 25/34 patientes avec demande de sérologie chlamydiae l’ont réalisée. La sérologie chlamydiae était positive dans 44% des cas. Seulement 25/33 patientes avec demande de recherche de mycoplasme l’ont réalisée. Le mycoplasme était positif dans 64% des cas. Il s’agissait de *Mycoplasma hominis* (43,8%) et de *Ureaplasma urealyticum* (81,3%). Le dosage des ßhCG plasmatiques était fait chez 17 patientes. Le test était positif dans 41,2% des cas. Le test urinaire des ßhCG était fait chez 8 patientes et 4 avaient un résultat positif. Une infection urinaire était confirmée chez 3/10 patientes (8%) due soit à *Escherichia coli* ou *Staphylococcus* aureus.

L’étiologie de la douleur était retrouvée chez 120 patientes (96%). Elle était d’origine génitale dans 95% des cas. Les causes étaient: les infections génitales hautes (IGH) aiguës soit 46 patientes (36,8%), les grossesses extra-utérines soit 23 patientes (18,4%) et les avortements soit 19 patientes (15,2%). Le traitement était surtout médical (92,8%) et chirurgical (20%). Les infections génitales hautes étaient traitées médicalement. Ce traitement associait les antibiotiques (65,5%), les anti-inflammatoires (56,9%) et les antalgiques (39,7%). Les avortements étaient traités surtout médicalement avec peu de cas de révision utérine. Des 23 cas de GEU, 5 étaient non rompues et traitées médicalement au méthotrexate. On avait 12 GEU rompues traitées par laparotomie et les 7 autres GEU non rompues étaient soit opérées par laparotomie (04 cas) soit par cœliochirurgie (03 cas). La douleur était encore présente chez 7 patientes 72 heures après l’institution du traitement. La régression de la douleur était favorable dans 99% des cas.

## Discussion

La prévalence des algies pelviennes aiguës était de 2,11% dans cette étude. Notre prévalence est inférieure à celle retrouvée par Randriamiarisoa *et al*. en 2000 [3]. La différence est due à la population d’étude qui dans leur étude était constituée de femmes enceintes uniquement contrairement à la nôtre où nous avons recensé toutes les femmes à l’exclusion des celles au troisième trimestre de grossesse. L’âge moyen des patientes était de 29,5 ± 6,9 ans. Il s’agit d’un réel problème dans notre milieu qui affecte les jeunes, les nullipares et les primipares. Nos résultats sont en accord avec la littérature parcourue sur le sujet [2,5-7]. Les étiologies des APA étaient celles couramment incriminées en période de vie sexuelle active (GEU, IGH, adhérences pelviennes, complication de grossesse etc.) ceci expliquerait la similitude d’âge et de parité entre notre étude et les séries retrouvées dans la littérature [5].

La douleur était insidieuse au début (62%), diffuse (56%) due à la prédominance des IGH (36,8%). Ceci est en accord avec Judlin *et al*. selon qui, le début insidieux de la douleur et le caractère unilatéral orientent vers une IGH [8]. La durée moyenne de la douleur était de 7 jours. Nos résultats sont semblables à ceux de la littérature indiquant qu’une évolution de plus de 4 jours est due à une infection génitale haute (IGH) [9]. Le caractère unilatéral est propre aux GEU et aux kystes ovariens dans les algies pelviennes aiguës. En cas d’hémopéritoine après la rupture, la douleur devient diffuse. Le saignement vaginal était associé à la douleur dans 39,2% des cas et pouvait être du à chacune des étiologies majeures d’APA dans notre étude. La douleur à la mobilisation utérine retrouvée à 61,6% traduit une irritation péritonéale du cul de sac de Douglas par le sang, le pus ou le liquide de kyste. En cas d’IGH, cette douleur est le critère majeur du diagnostic clinique [10, 11]. Elle est retrouvée dans 25% des cas des GEU et des kystes en rapport avec un hémopéritoine [1].

L’échographie pelvienne était pratiquée dans 87,2% des cas (Tableau 2), le coût faible, le caractère non invasif et de réalisation facile justifieraient cette forte utilisation. Nos résultats sont proches des recommandations de certains auteurs [12], qui préconisent la pratique systématique de l’échographie en cas d’algies pelviennes aiguës. Par contre, seulement 5 patientes ont bénéficié de cœliochirurgie, technique de référence dans le diagnostic et le traitement des algies pelviennes aiguës [13, 14]. Le coût élevé de la cœlioscopie (500.000 francs CFA, ou $ 1.000 USD) pourrait justifier cette faible utilisation de la cœlioscopie.

**Tableau 2:** Répartition des algies pelviennes aiguës selon les examens morphologiques

Exploration de la douleur	Algies pelviennes aigues N=125
	Fréquence	%
**Echographie pelvienne**	**109**	**87,2**
Normale	11	10,1
Inflammation pelvienne	31	28,4
GEU	21	19,3
Kyste ovarien	17	15,6
Avortement	10	9,2
Autres*	30	27,5
**Cœlioscopie**	**5**	**4,0**
Torsion d’annexe	2	-
Adhérence pelvienne	2	-
GEU	3	-
Kyste ovarien	1	-
Utérus Gravide	1	-

* Grossesse normale, myomes utérins, hydrométrie, abcès pelvien, dystrophie ovarienne, avortement incomplet, polype endométrial

Dans 96% des cas, l’étiologie de la douleur était retrouvée (Tableau 2). L’origine génitale était prédominante (95%). Nos résultats sont en accord avec ceux de Kurt *et al*. qui avaient retrouvé les causes gynécologiques dans 93,2% comme étiologie des algies pelviennes aiguës [6]. Le Service de Gynécologie-Obstétrique a servi de lieu de recrutement. Il reçoit les femmes qui ont des plaintes gynécologiques. Ceci pourrait justifier la prédominance des causes gynécologiques. Les IGH étaient les premières causes génitales des algies pelviennes aiguës (36,8%) et la population avait un âge moyen jeune dans notre étude. nos résultats corrobore avec certains auteurs qui justifient cette prédominance des IGH chez les jeunes par les comportements à risque vis-à-vis des infections sexuellement transmissibles [15, 16]. Dans l’étude de Kontoravdis *et al*. en Grèce, les IGH étaient retrouvées comme étiologies principale des APA soit 22,8%. Par contre, nos résultats diffèrent de ceux de Randriamiarisoa *et al*. à Madagascar [7], où l’avortement était prépondérant (59,7%), mais son étude était réalisée uniquement chez les femmes enceintes dans une maternité. Dans le même sens, Fauconnier *et al*. en France [5], rapportait lui aussi les complications de la grossesse intra-utérine comme étiologie majeure des APA dans 40,7%. Il y avait une prépondérance des femmes enceintes dans la population étudiée (48,6%). D’autres auteurs comme Anteby *et al*. en Israël [13], Kurt *et al*. en Iran [7] avaient trouvé une prédominance des kystes ovariens dans 27% et 50% respectivement. Par contre, chez Morino *et al*. en Italie, aucun diagnostic n’était retrouvé dans la plupart des cas (37%) [17, 18]. Le traitement médical primait (92,8%) sur la chirurgie (20%). La prédominance des IGH et le coût élevé de la cœlioscopie pourraient expliquer la prédominance du traitement médical.

## Conclusion

Les algies pelviennes aiguës constituent un motif de consultation fréquent en gynécologie. Les causes majeures sont les infections génitales hautes, la grossesse extra-utérine et l’avortement. Quand il s’agit de grossesse extra-utérine, le traitement est le plus souvent chirurgical utilisant surtout la laparotomie. Nous recommandons qu’une étude soit menée avec un échantillon plus grand pour en déterminer la prévalence et généraliser les résultats.

### Etat des connaissances actuelles sur le sujet

- Symptôme fréquent en consultation d’urgence gynécologique dont la prévalence exacte n’est pas connue;
- Variétés de causes sous-jacentes gravidiques que non gravidiques, en contexte non gravidique, les causes peuvent être gynécologiques ou non;
- La détermination du contexte gravidique ou non est essentielle pour affiner les hypothèses diagnostiques.

### Contribution de notre étude à la connaissance

- Nous avons pu déterminer la prévalence des algies pelviennes dans notre service;
- Les principales causes en contexte non gravidique chez nous sont dominées par les infections génitales hautes et en contexte gravidique c’est la grossesse extra-utérine et les avortements qui dominent le tableau;
- Le traitement de la grossesse extra-utérine demeure la laparotomie.
